# Sputum induction and its diagnostic applications in inflammatory airway disorders: a review

**DOI:** 10.3389/falgy.2023.1282782

**Published:** 2023-10-12

**Authors:** Beatriz Goncalves, Ukpai A. Eze

**Affiliations:** ^1^ NIHR Leicester Biomedical Research Centre, Department of Respiratory Sciences, Glenfield Hospital, Leicester, United Kingdom; ^2^ Leicester School of Allied Health Sciences, Faculty of Health and Life Sciences, De Montfort University, Leicester, United Kingdom

**Keywords:** sputum induction, spirometry, asthma, chronic obstructive pulmonary disease, dithiothreitol sputum processing, airway inflammatory disorders, induced sputum cytospins, sputum eosinophil differential cell count

## Abstract

Sputum induction is a technique that covers the induction and the subsequent processing of the expectorate primarily for the analysis of cells and different inflammatory biomarkers present in the airways to further understand the pathophysiology of different inflammatory respiratory disorders such as asthma and chronic obstructive pulmonary disease (COPD) as well as the diagnosis of lung diseases such as lung cancer, tuberculosis, and *Pneumocystis jirovecii* pneumonia. It is a non-invasive, safe, cost-effective, and reliable technique reported to exhibit a high success rate. However, due to being technically demanding and time-consuming and having the need of employing trained staff, this technique is only used in restricted research centres and in limited centres of clinical use. When the sputum is collected after induction, the primary goal is to obtain a differential cell count and evaluate the molecular biomarkers of airway inflammation such as eosinophil cationic protein, eosinophil-derived neurotoxin, major basic protein, tryptase, cytokine production [e.g., interleukin (IL)-5], albumin, and fibrinogen. In addition, cytospins from the processed sputum are used for immunocytochemical staining of cellular products such as EG-2 reactive protein, granulocyte-macrophage colony-stimulating factor, tumour necrosis factor alpha, and IL-8 that play significant roles in understanding the pathophysiology of inflammatory airway diseases. Nowadays, this technique can be further used by performing an additional analysis such as flow cytometry and *in situ* hybridisation on the sputum supernatant to investigate more the immune response and pathophysiological process of such various respiratory diseases. In addition, the application of sputum fluid phase to assess the biomarkers could be used more routinely in pathological laboratories for diagnosing lung cancer, COPD, and asthma as well as for monitoring lung cancer progression and asthma and COPD treatment, allowing for early detection and a better treatment provided by the clinicians.

## Introduction

Airway diseases are a major problem in today's society that are significantly increasing due to climate change and affecting the lives of many individuals around the world and the healthcare services (1). The main airway diseases include asthma and chronic obstructive pulmonary disease (COPD). Both diseases are characterised with exacerbations and airway remodelling. Most importantly, in both pathologies, airway inflammation is considered their primary cause. It has been shown that improved understanding, management, and treatment of these respiratory diseases lie with the measurement of airway inflammation and, in some cases, the associated microbial infections (1–3).

Several methods to study airway inflammation, namely, direct methods such as bronchoalveolar lavage (BAL) and bronchial biopsies and indirect methods such as blood analysis, lung function tests, and even symptom assessment, were identified (4). On the one hand, the direct methods are most commonly used compared with the indirect ones as these techniques generate more reliable results. However, they are invasive, expensive procedures, and they cannot be repeatedly performed due to its invasive nature; therefore, it is not feasible to be performed in large-scale clinical studies (5). In addition, both methods sample different parts of the airways. Bronchoscopy samples cells and mediators that are present in the lumen of the airways and therefore enable the mucosal tissue to be biopsied. BAL mainly allows the distal part of the bronchus to be sampled. However, mixing of the different lung compartments can also occur, which do not allow an accurate result of the sample obtained. Furthermore, the mediators and cells obtained in the samples are most often diluted in large amounts of saline, and blood contamination in this procedure can also occur. On the other hand, the indirect methods are inexpensive and not invasive. However, the results obtained using these techniques do not correlate well with the direct assessment of airway inflammation (4, 5).

Due to the limitations of these techniques, another direct measurement of airway inflammation has been developed, called sputum collection. Sputum collection involves sputum production either spontaneously or by induction and subsequent processing of the sputum. Spontaneous sputum was routinely used in the past. However, research showed that most of the samples obtained were of poor quality and not every patient was able to produce sputum (6–9). In 1958, Bickerman et al. (10) first used sputum induction for diagnosing lung cancer by making patients inhale hypertonic saline to produce sputum in order to overcome this limitation. Then, later in 1986, Pitchenik et al. (11) used the same technique to diagnose *Pneumocystis carinii* (now *Pneumocystis jirovecii*) pneumonia in patients that were infected with HIV and in patients with AIDS.

Pin et al. (12) published the first study of sputum induction using hypertonic saline as a method to study airway inflammation in patients with asthma, and since this first successful attempt, different researchers have reported using sputum induction to study airway inflammation not only in asthma but also in other respiratory disorders such as COPD and chronic cough (9, 13). For the wide use of these techniques and for the comparison of the results of the different published papers, this method became standardised in 1999 by the task force that was approved by the European Respiratory Society (13). Standardisation of this technique not only enabled data to be globally compared but also allowed the quality and the reproducibility of sputum samples to be improved (14). Consequently, allowing sputum induction and its successive processing to become an important non-invasive research and clinical tool for the assessment of airway inflammation and for the discovery of novel new therapeutics.

Sputum induction is non-invasive and less costly compared with the other techniques (15). In addition, it can be performed as required and repeatedly in patients regardless of the severity of their disease and even during exacerbations, making this technique appropriate for large studies and clinical trials with multiple visits. Although it can cause bronchospasm in patients with hyperresponsive airway, this can be overcome if patients are given a short-acting beta-agonist before the technique. It has been demonstrated that sputum induction is a safe, successful, reproducible, and reliable method, making it a useful tool in the assessment of airway inflammation (6–9, 14, 16).

Generally, sputum expectoration is induced; therefore, it can be collected and processed to obtain a differential cell count and subsequently measure the type of inflammation present in the lumen of the airways. Different methods currently used in literature include the following: plug selection method, whole sputum method, phosphate-buffered saline (PBS)-treated method, and traditional sputum processing method using dithiothreitol (DTT). This paper reviews the different methodology for sputum induction and laboratory processing in literature and the different diagnostic applications of sputum induction.

## Sputum induction

Sputum induction is performed by inhalation of isotonic or hypertonic saline solution using ultrasonic nebulizers which is followed by the expectoration of airway secretions by coughing (Table 1). Before this technique is performed, the lung function of the patient needs to be measured using a spirometry. This needs to be conducted due to the fact that hypertonic saline inhalation by asthmatic patients causes bronchoconstriction (17). Spirometry is used over measuring peak flow because they offer greater sensitivity of forced expiratory volume in 1 s (FEV_1_) to detect bronchoconstriction. It is recommended that short-acting beta-2-agonist should always be administered as a standard protocol before treatment (18). The induction must be conducted under medical supervision, performed by trained technicians, and a physician needs to be always present during the process in case any adverse events occur during the procedure. Approximately 200–400 µg of salbutamol is given using a pressurised metered-dose inhaler (pMDI) as pre-treatment to all the patients as broncho-protection against saline causing no affect in cell counts and inflammatory markers (19).

**Table 1:** Summary of selected studies on different methods of sputum induction.

References	Method of sputum induction used	Outcome
Pavia et al. (20)	To induce sputum, this study used hypertonic saline as opposed to using isotonic saline.	The use of hypertonic saline resulted in fast and enhanced clearance of the whole lung, and the mean weight of the sputum was higher compared with the control.
Iredale et al. (21)	4.5% hypertonic saline was used in this study.	The use of hypertonic saline was successful in inducing sputum and obtaining a good sputum sample. No correlation between eosinophilia of induced sputum and bronchial responsiveness to inhaled hypertonic saline.
Popov et al. (22)	This study used both hypertonic saline and isotonic saline.	Hypertonic saline was shown to be more successful in inducing sputum than isotonic saline. In addition, hypertonic saline resulted in an average weight of sputum superior to the normal saline due to the sputum being more easily induced. The cell viability, the differential cell count, and the number of cells in the sputum obtained using both types of saline were very similar. No statistical difference was found between the subjects. Normal saline was shown to cause less discomfort.
Bacci et al. (23)	Both hypertonic saline and isotonic saline were used in different patients to investigate their differences.	Showed that no significant difference was noted in the percentage of inflammatory cells produced by either isotonic or hypertonic saline. Isotonic saline-induced sputum showed greater squamous cell percentage due to more saliva contamination. No difference was reported in sputum eosinophil percentages. Hypertonic saline caused significant bronchoconstriction in 18 patients, and it increased bronchial hyperresponsiveness. The isotonic saline is as successful in inducing sputum as hypertonic saline.
Loh et al. (24)	Both types of saline were investigated in this study.	Higher total sputum cell counts and higher cell viability were shown using hypertonic saline compared with using isotonic saline. Hypertonic saline also showed less squamous cell contamination. No significant difference was found between the number of macrophages, neutrophils, and lymphocytes in the sputum produced using both isotonic and hypertonic saline. In conclusion, this study showed that hypertonic saline is more effective than isotonic saline, but it is less well tolerated.

**Figure 1:**
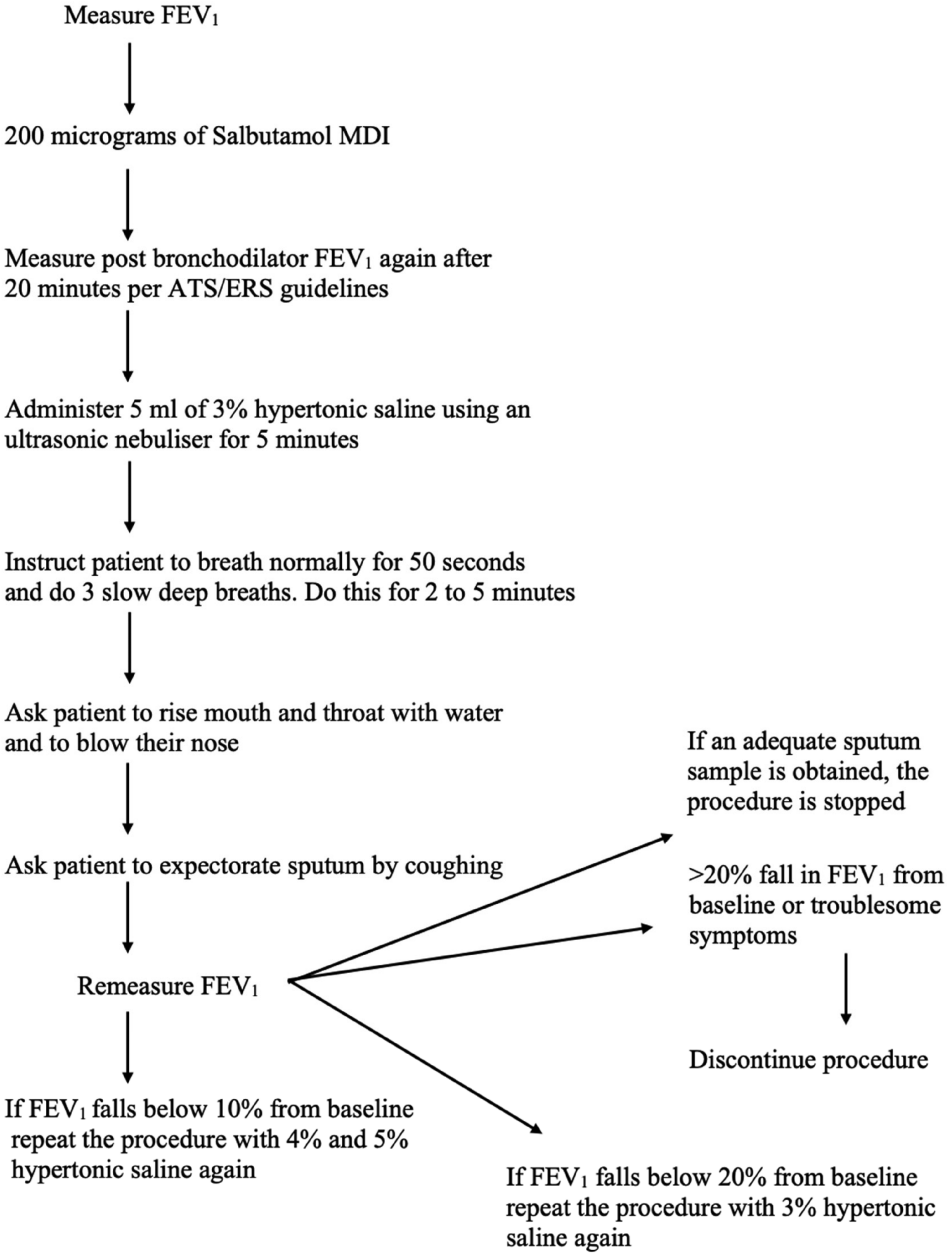
Overview of sputum induction protocol.

Saline concentrations may vary from 0.9% to 7% in different studies. However, 4.5% of sodium chloride concentration is the recommended value for general use (Figure 1). One study showed that no significant difference was found in the cellular composition and in the differential cell count of the induced sputum when using isotonic or hypertonic saline for the induction (25). However, different research showed that hypertonic saline is more effective in inducing sputum (26). During the procedure, different parts of the airways are sampled; firstly, the central airway, then the peripheral airway, and lastly the alveoli are sampled, and this occurs during different points of the induction. Shorter durations (15–20 min) and longer periods (about 30 min) of inhalation seem to exhibit the same success rate and feasibility in sputum production (26). However, Chanez et al. stated that the consensus is to use a cumulative duration of 15–20 min of nebulisation with the patient being asked to cough and expectorate every 5 min and in each period the lung function being measured for detection of bronchoconstriction (27).

## Induced sputum processing

**Figure 2:**
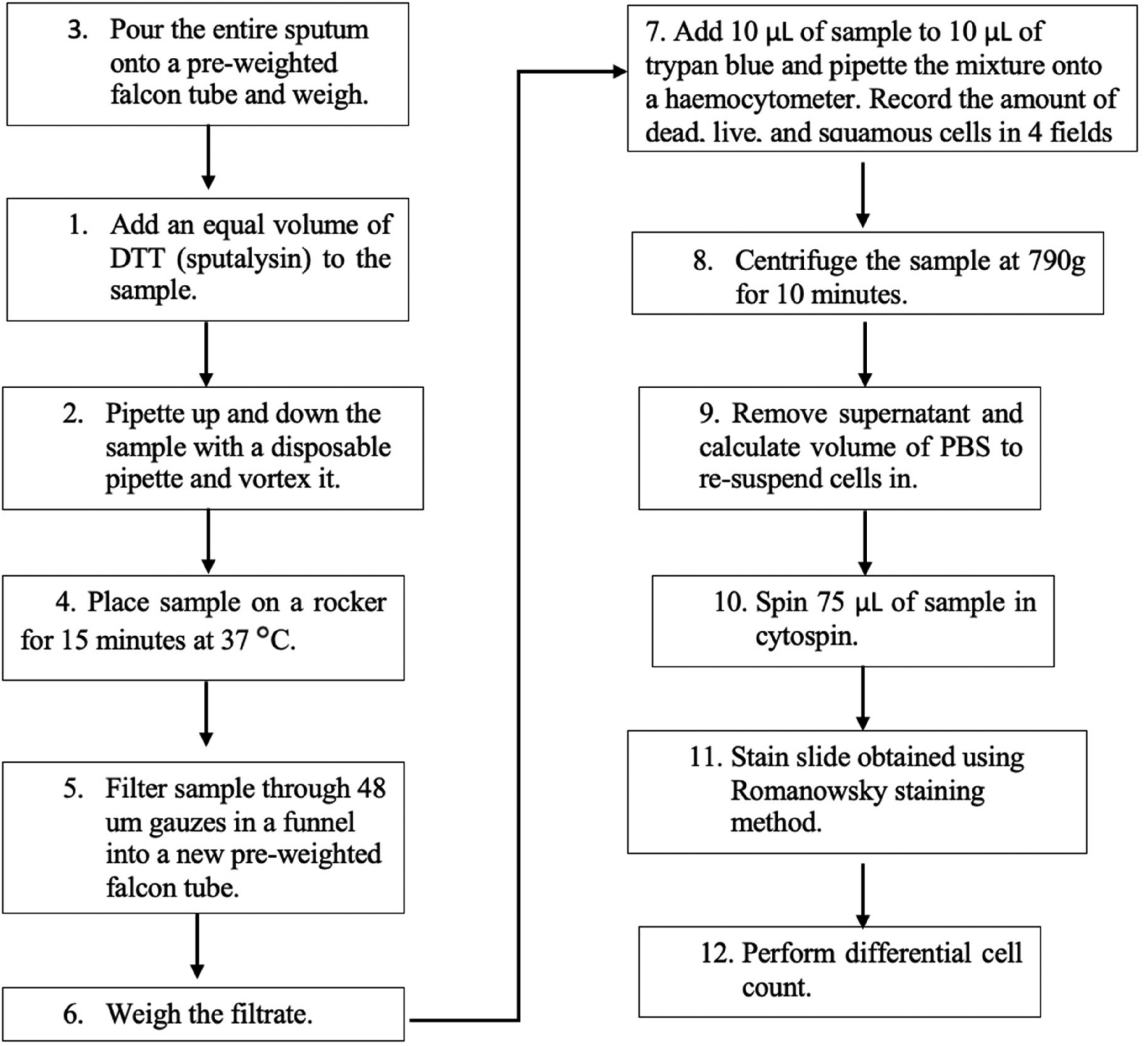
Whole sputum (non-phosphate buffered saline) method.

**Figure 3:**
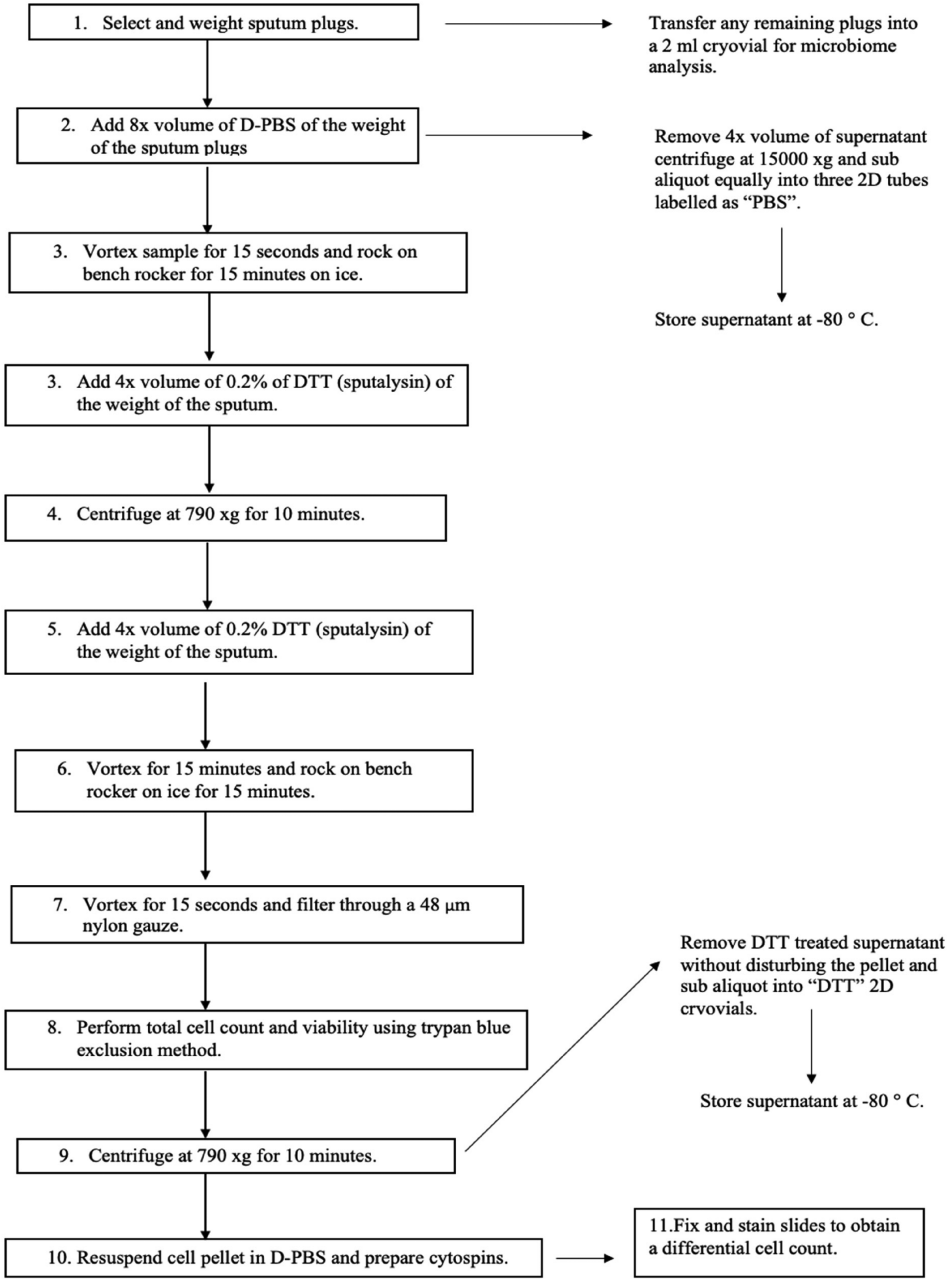
Selected sputum phosphate buffered saline method.

An expert consensus-based recommendation was published in 2002 by the European Respiratory Task Force (ERTF) in which they formulate the basic rules for the use of sputum processing technique in adults and in children (13). Two main protocols are used in literature for processing the expectorate: the whole sputum method (Figure 2) and the selected plug method (Figure 3); both of these methods are shown to be valid and reproducible by various studies (22, 28). However, a deviation of these methods not related to how the sputum is selected (whole or plugs) but on how the sputum is treated after selection has been developed: the PBS method and the non-PBS method.

The whole sputum processing method has been first described by Fahy et al. (28), and it comprises the selection of all sputum samples, such as selection of the mucus plugs that are opaque and dense and the clear surrounding saliva. On the other hand, the plug selection method, described by Popov et al. (22), is the selection of only the opaque or coloured mucus plugs for the whole sputum sample to reduce saliva contamination. The methods may differ on how the sputum sample is selected; however, the rest of the protocol is the same for both if the non-PBS method is performed. The sputum needs to be processed for no longer than 3 h of the sample being collected to ensure that the cell viability is maintained. DTT is used to treat the sample to allow complete homogenisation and to ensure that cells are released and dispersed (Figure 2). In the non-PBS method, DTT is used first, but in the PBS method, the PBS is used first to wash the cells to obtain a supernatant that is further employed for the analysis of various inflammatory mediators such as interleukins present in the supernatant (29). The cellular and biochemical analysis of the sputum is conducted in research using the PBS method; however, in the clinical setting, the non-PBS method that does not collect any supernatants is used (13).

Centrifugation is needed in both methods to obtain the supernatant, PBS and DTT, for the analysis of the sputum; different studies used different centrifugal forces, but they all range from 300 ×g to 1,500 ×g and last for about 10 min (13). Different studies use different stains for differential cell counts, but all have in common in the determination of non-squamous cells, meaning the counting of eosinophils, neutrophils, macrophages, and lymphocytes. Spanevello et al*.* (30) showed that the method of selecting sputum plugs is a more advantageous technique over the whole sputum sample as the cell viability percentage, total cell count percentage, is higher than the whole sputum method. However, it was also demonstrated that both methods have the same ability to distinguish individuals with asthma from healthy individuals (30, 31). The whole sputum method provides a faster processing compared with the selected method, but the saliva contamination in the whole sputum samples causes an increase in squamous cell contamination that decreases the quality of the sample of the cytospins (12, 31). A limitation of the selected sputum method is that not all samples obtained will contain sputum plugs that cause a limitation in the sample processing, and the plugs are not representative of all the samples.

## Applications of induced sputum

Induced sputum has been clinically used in a variety of different ways, such as the detection of lung cancer, management of asthma in asthmatic patients by observing changes in sputum eosinophils, phenotyping of different types of asthma, and assessment of airway inflammation in COPD patients (Table 2). In asthma, this technique has been used as a diagnostic tool by performing sputum eosinophil count as asthma is an airway disease associated with sputum eosinophilia. In addition, induced sputum has enabled the assessment of short-term and long-term response of patients with asthma to inhaled corticosteroids providing evidence that corticosteroids have a moderate impact on improving sputum eosinophils and symptoms (8, 32). This finding allowed the use of induced sputum for the assessment of airway inflammation in response to different drugs and therefore allowed for a more personalised treatment as different phenotypes of asthma respond differently to different medications. Cytospins from processed sputum are used for immunocytochemical staining of cellular products such as EG-2 reactive protein, granulocyte-macrophage colony-stimulating factor (GM-CSF), tumour necrosis factor alpha (TNF-α), and interleukin (IL)-8 (8, 33). Similarly, the supernatants obtained from the processed sputum are used in evaluating molecular markers of airway inflammation such as eosinophil cationic protein (ECP), eosinophil-derived neurotoxin, and major basic protein for eosinophil activation, tryptase for mast cell activation, and cytokine production (e.g., IL-5) as well as albumin and fibrinogen that are useful markers of microvascular leakage (6, 8). The levels of these molecular markers were raised in the processed sputum from asthmatic patients than from control subjects (3, 6, 33–35).

**Table 2:** Summary of different papers utilising sputum induction and its major outcomes.

Reference	Induced sputum application	Major outcomes
Neumann et al. (50)	Sputum induction can be used as a non-invasive method for detecting lung cancer.	This study assessed the frequency of premalignant and malignant cells in the sputum of lung cancer patients. Out of 444 patients, 74.6% of the expectorate provided by the patients were positive for premalignant or worse cells, and 48.7% of the sputum was positive for only malignant cells. This shows that sputum cytology can be used as a diagnostic tool for lung cancer detection.
Kelsen et al. (51)	Sputum induction can be used in part of clinical trials. In this particular study, the efficacy and safety of astegolimab in asthmatic patients was investigated.	Astegolimab blocks IL-33 signalling by targeting an IL-33 receptor in both asthmatic patients with and without high eosinophils. This study found that astegolimab is a safe and well-tolerated drug that reduced frequent exacerbations by blocking IL-33/ST2 pathway.
Diver et al. (52)	Microbiome obtained from sputum samples can be subjected to further analysis. In this study, the microbiome of the asthmatic patients and COPD patients has been studied if sputum microbiomic clusters exist in stable airway diseases and therefore they can be differentiated.	This study identified two clusters, *Haemophilus-*high and *Haemophilus-*low, and these clusters can be found by the γProteobacteria/*Firmicutes* (γP/F) ratio.

Correspondingly, Eltboli and Brightling (36) showed that in COPD patients, induced sputum can be used to assess airway inflammation for individuals suffering from the disease by performing differential cell counts and therefore allowing for a more tailored treatment and management and in diagnosing chronic lung diseases. A raised sputum neutrophilia combined with high sputum levels of TNF-α and IL-8 has been linked with COPD and is suggested as a potential marker for the diagnosis of COPD (2, 37). Kirsch et al. (38) showed that the analysis of induced sputum can be used in diagnosing *Pneumocystis carinii* (currently named *Pneumocystis jirovecii*) pneumonia (38, 39). In addition, induced sputum can be used as a diagnostic tool in detecting pulmonary tuberculosis (40–43), pulmonary sarcoidosis (44, 45), and lung cancer (46–49). Therefore, the introduction of this method in pathological laboratories will provide an additional tool for diagnosing different pulmonary diseases (53).

In research, several techniques can be applied on induced sputum such as DNA extraction on microbiome obtained for the sputum sample, enzyme-linked immunosorbent assay (ELISA), flow cytometry, and *in situ* hybridisation to further understand the underlying airway inflammation and the role of different cells and cytokines in different airway diseases for better treatment and management. Furthermore, induced sputum has been used in various clinical trials to test new drugs for the treatment of asthma. In a study by Russell and Brightling (54), sputum induction was used as a method to support in the development of a new therapy (mepolizumab) that targets IL-5 to help with asthma exacerbations. The emergence of proteomics, lipidomics, metabolomics, exosomics, exposomics, transcriptomics, functional assays, whole genome sequencing, genome-wide sequencing, microRNA assays, and bioinformatics tools (55) as well as data science, artificial intelligence, and machine learning (52) will further revolutionalise the usefulness of induced sputum as both a diagnostic and research tool in understanding the pathophysiology and diagnosis and for monitoring the treatment progress of asthma, COPD, lung cancer, and pulmonary tuberculosis.

## Concluding remarks

As discussed in the previous sections, sputum induction and processing is a well-tolerated, safe, and non-invasive method for the collection and analysis of cells from the airways making it an important procedure for the diagnosis of various respiratory diseases such as asthma, COPD, chronic cough, or idiopathic pulmonary fibrosis. However, the technique is currently limited to research services and specialised centres in clinical practice because it is technically demanding and time-consuming and requires trained staff. In the laboratory in which BG performed her placement, the procedure is generally used for the administration of research and clinical trials. A patient is initially prepared, and spirometry [forced expiratory volume in 1 s (FEV_1_)] is performed according to the standard criteria formulated by the American Thoracic Society (ATS)/European Respiratory Society (ERS) (13). Sputum is induced by inhalation of 3% hypertonic saline solution (for patients with post-bronchodilator FEV_1_ of >65% predicted) or 0.9% isotonic saline (for patients with post-bronchodilator FEV_1_ of ≤65% predicted) using an ultrasonic nebuliser after the administration of 400 µg of inhaled salbutamol from a metered-dose inhaler (MDI) via a spacer or an equivalent dose of other inhaled short-acting β2-agonist. The nebulisation and sputum collection are performed under medical supervision. Sputum samples are collected on ice and processed following the procedure in Figure 3 within 2 h of expectoration to ensure optimum cell viability. However, at the peak of the COVID-19 pandemic, spontaneously expectorated sputum was mainly used during a stable clinical state for the health and safety of the research administrators and other patients since sputum induction posed a major risk of increasing aerosol transmission.

In a recent prospective internally controlled interventional trial carried out at the Children's Hospital for Wales (Cardiff, UK) in children with cystic fibrosis, it was reported that sputum induction is superior to cough swab for pathogen detection, is effective at sampling the lower airway, and is a credible surrogate for bronchoalveolar lavage in symptomatic children (56). In the study, 124 patients were prospectively recruited, and 84% of the sputum induction were successful; the sputum induction procedures was well tolerated by the patients (56). In a study by Guiot et al. (5), the success rate of sputum induction and a readable cytospin was 75% in healthy subjects, 82% in patients with asthma, and 82% in COPD patients, with an overall success rate of 82%. In addition, Pin et al. (12) reported a success rate of 77% in the first attempts and 84% in the second attempts among 17 asthmatic and 17 healthy patients. Other researchers have reported success rates of 80%–91% in adults and children (57–59), 81% in healthy adult subjects (60), and 80% in patients with asthma (61). However, Vieira et al. (62) reported that sputum induction in patients with severe exacerbations of asthma using a modified method was successful in 93% of subjects. In their study, Vieira et al. (62) performed sputum induction in 45 patients after pre-treatment with 400 mg salbutamol by inhalation, for repeated periods of 1–2 min, of an aerosol of isotonic saline only or followed by hypertonic (3%–4%) saline. They recommended that sputum induction can be successful and safe even in severe exacerbations of asthma if this modified method is carefully followed.

Although sputum induction is generally regarded as safe and successful in both children and adults (63–70), recommended contraindications that must be taken into account to preclude or delay induced sputum collection are noted (Table 3). After exclusion or resolution of the contraindicated conditions, sputum induction can be considered (71). Similarly, at the peak of the COVID-19 pandemic caused by severe acute respiratory syndrome coronavirus 2 (SARS-CoV-2), a multidisciplinary consensus on sputum induction biosafety was issued since sputum induction is associated with the generation of aerosols, cough manoeuvres, and the handling of sputum samples (73). The conditions shown in Table 4 were regarded as contraindications for sputum induction during the COVID-19 pandemic.

**Table 3:** Contraindications for sputum induction in the non-pandemic period (71, 72).

**Absolute contraindications**
As hypertonic saline which is commonly used in sputum induction causes bronchoconstriction, the procedure should only be performed after pre-medication with salbutamol and under medical supervision in patients with asthma, suspected asthma, or severely impaired lung function (FEV_1_ < 1.0 L).
**Relative contraindications**
Sputum induction causes severe coughing, and therefore the procedure should not be performed in patients in whom severe coughing may be harmful. This may include patients with:
• Haemoptysis of unknown origin • Acute respiratory distress • Unstable cardiovascular status (e.g., arrhythmias, angina) • Thoracic, abdominal, or cerebral aneurysms • Hypoxia (saturated oxygen level of less than 90% on room air) • Lung function impairment (FEV_1_ < 1.0 L) • Pneumothorax • Pulmonary emboli • Fractured ribs or other chest trauma • Recent eye surgery, face deformity, or history of recurrent nosebleed • History of recurrent convulsion • Patients who have cognitive impairment and are unable to follow instructions

**Table 4:** Contraindications for sputum induction during the COVID-19 pandemic (73).

**Absolute contraindications**
• Suspected or diagnosed SARS-CoV-2 infection • SARS-CoV-2 pandemic peak period • Exacerbation episode (whether or not infectious) in the previous 4 weeks • Active smoking on the day of the test • FEV_1 _< 600 ml
**Relative contraindications**
• FEV_1_ < 50% or <1.0 L. • High probability of bronchoconstriction: very positive bronchodilator test, positive low-dose bronchoprovocation test, overuse of rescue medication

However, it has been recommended that sputum induction by the inhalation of lower concentration of the isotonic saline, hypertonic saline, or dry mannitol powder using an ultrasonic nebulizer are safe for patients with relative contraindications (73). In a comparative assessment of the use of hypertonic (4.5%) saline and a mannitol in 55 subjects with stable asthma, induced sputum was successfully obtained from 49 (89%) subjects using the hypertonic saline solution and 42 (76%) subjects challenged with mannitol solution (74). In a different clinical trial consisting of 592 participants, it was demonstrated that mannitol dry powder had a sensitivity of 81% and a specificity of 87% with respect to a cut-off of 15% fall in FEV_1_ for 4.5% hypertonic saline (75). It was further demonstrated that inhalation of dry mannitol powder had a sensitivity of up to 89% identifying the presence of asthma and a specificity of 95% for clinical diagnosis of asthma (75). A previous study had also shown a correlation between 15% fall in FEV_1_ for mannitol and hypertonic saline (76). This further corroborates the safety of sputum induction using the inhaled mannitol powder and hypertonic saline in detecting airway hyperresponsiveness, in obtaining good-quality sputum for the analysis of inflammatory cells and inflammatory mediators, and for predicting the inflammatory phenotype in individual patients with asthma (73–77). It is worth noting that well-trained staff in hospitals can successfully perform sputum induction with good-quality sputum for diagnostic applications; therefore, readers are encouraged to conduct sputum induction as this is now generally considered safe and useful results obtained for the management of patients with airway disorders.

Sputum induction and the subsequent processing of sputum are useful methods to assess the airways and have various applications from diagnosis to developing more target therapies compared with other methods. Even with limitations and with only few laboratories that are able to use this technique due to being time-consuming and having the need of employing trained workforce, it should be made more accessible and be put more into a clinical setting by all medical centres. In the future, sputum induction can be used more widely in research to provide further information on the mechanisms, both cellular and molecular, of the different airway diseases, so that the treatment and management is even more specific towards different patients and therefore more effective than the treatments currently available. In addition, the use of sputum fluid phase in the assessment of biomarkers could be used more routinely in pathological laboratories for the diagnosis of lung cancer and for monitoring cancer progression, allowing for early detection and a better treatment provided by the clinicians.
